# MoEformer: An Input-Adaptive Multi-Scale Mixture-of-Experts Transformer for Automatic Modulation Recognition

**DOI:** 10.3390/s26217045

**Published:** 2026-11-07

**Authors:** Jiale Wang, Wupeng Xie, Yaxin Mu

**Affiliations:** 1 School of Information and Communication Engineering, Beijing Information Science and Technology University, Beijing 100101, China; jiale.wang@bistu.edu.cn (J.W.); muyaxin15@mails.ucas.ac.cn (Y.M.); 2 Artificial Intelligence Institute of China Electronics Technology Group Corporation, Beijing 100041, China

**Keywords:** automatic modulation recognition, mixture-of-experts, Transformer, deep learning

## Abstract

Automatic modulation recognition (AMR) is essential for spectrum sensing and non-cooperative wireless monitoring. Although multi-scale temporal representations provide complementary information from received in-phase and quadrature (I/Q) signals, their fusion faces two challenges: mismatched feature sequence lengths and the inability of fixed fusion rules to adapt scale contributions to individual inputs. To address these challenges, we propose MoEformer, a multi-scale Mixture-of-Experts Transformer with input adaptability for AMR from raw I/Q sequences. Parallel experts extract scale-specific features from temporally resampled inputs. Frequency-domain feature alignment maps the expert features to a common sequence length, while a learned gating network assigns sample-dependent weights before Transformer-based temporal modeling and attention pooling. Experiments on RadioML2016.10a, RadioML2016.10b, and RadioML2018.01A achieve overall classification accuracies of 63.74%, 66.24%, and 64.22%, respectively, outperforming all compared baselines. For SNR from −20 to 0 dB, MoEformer exceeds the strongest baseline on each dataset by 1.07, 1.56, and 1.43 percentage points, respectively. Ablation studies support the contributions of adaptive expert weighting and frequency-domain feature alignment, while the additional inference overhead highlights the trade-off between recognition accuracy and computational efficiency.

## 1. Introduction

The rapid growth of IoT devices and heterogeneous wireless services has increased the demand for intelligent spectrum awareness. Automatic Modulation Recognition (AMR), a key physical-layer technology in cognitive and non-cooperative communication systems, identifies the modulation format of received signals without prior knowledge of the transmitter or channel state [1]. It supports applications such as spectrum monitoring, dynamic spectrum access, and communication surveillance [2]. Therefore, robust and computationally efficient AMR is increasingly important in dynamic wireless environments.

### 1.1. Related Work

Early AMR studies mainly followed two classical paradigms: likelihood-based (LB) and feature-based (FB) methods [3]. LB approaches perform recognition through statistical hypothesis testing and can achieve strong theoretical performance under ideal assumptions [4]; however, their practical deployment is limited by high computational complexity and the requirement for accurate prior knowledge of channel parameters, noise statistics, and signal models. In contrast, FB methods extract handcrafted descriptors, including instantaneous statistics [5], spectral features [6], and higher-order cumulants [7], followed by conventional classifiers such as decision trees and Support Vector Machines (SVMs) [8]. Nevertheless, their performance relies heavily on the quality and generalizability of manually designed features, making them vulnerable to low SNR, fading, synchronization impairments, and signal conditions beyond predefined assumptions [9]. These limitations have driven the development of data-driven approaches that directly learn modulation representations from received signals.

The rise of deep learning has substantially advanced AMR toward end-to-end representation learning, reducing the reliance on handcrafted feature engineering. Pioneering work by O’Shea et al. demonstrated that convolutional neural networks (CNNs) can learn discriminative representations directly from raw in-phase and quadrature (I/Q) sequences, establishing deep learning as a widely adopted paradigm for AMR [10,11]. CNN-based models exploit local receptive fields and weight sharing to extract hierarchical local patterns from received signals and have achieved competitive recognition performance on commonly used AMR benchmarks [12,13]. Residual connections were subsequently introduced to alleviate optimization degradation in deeper AMR networks, enabling ResNet-style architectures to provide improved recognition performance over corresponding convolutional baselines, particularly under relatively high-SNR conditions [14,15,16]. Other studies reformulated AMR as an image-classification problem by converting one-dimensional temporal signals into two-dimensional representations [17]. For example, Peng et al. [18] mapped received signals into constellation diagrams and employed classical convolutional backbones (such as AlexNet and GoogLeNet) to characterize clustering structures for distinguishing QAM signals. Subsequent studies further combined constellation representations with deep feature fusion and attention mechanisms to improve feature separability [19,20]. Time–frequency representations generated using short-time Fourier transform (STFT) [21] and wavelet transform [22] have also been adopted to incorporate transform-domain information. Although these representations can enhance feature separability, they introduce additional preprocessing overhead and may discard information contained in the original temporal waveform. More importantly, conventional CNN-based architectures rely on receptive fields largely determined by the predefined network structure, which limits their flexibility in modeling temporal dependencies distributed over different ranges [23].

To improve the modeling of long-range temporal dependencies, recurrent neural networks (RNNs), including long short-term memory networks (LSTMs) [24,25] and gated recurrent units (GRUs) [26,27], have been introduced into AMR and shown to effectively characterize sequential signal dynamics [28]. Nevertheless, recurrent computation inherently limits parallelism and can reduce computational efficiency for long observation sequences. Inspired by their wide applications in natural language processing [29], speech recognition [30], and computer vision [31], Transformer architectures [32] have therefore attracted increasing attention in AMR because self-attention enables highly parallelized modeling of long-range dependencies [33,34]. For example, MCformer [35] integrates a convolutional front-end and Transformer blocks to hierarchically extract fine-grained local temporal patterns and long-range global dependencies. With mixed-channel feature modeling, it effectively captures multi-granularity temporal correlations at different feature levels while maintaining low parameter complexity.

Taken together, existing AMR research has evolved from model-based and handcrafted-feature approaches toward deep representation learning and attention-based sequence modeling. Recent methods have further improved recognition performance by exploiting local waveform patterns, transformed-domain representations, long-range temporal dependencies, and multi-scale features.

### 1.2. Research Gap and Motivation

Despite these advances, existing multi-scale AMR architectures primarily focus on constructing multiple temporal representations, whereas the contribution of these representations is typically determined by fixed architectural rules or input-invariant fusion. Consequently, the same scale combination is applied to signals with different modulation types and channel conditions. Temporal resampling allows the same received signal to be represented at different resolutions, while scale-specific feature extractors can learn distinct representations from these inputs. Since the usefulness of such representations may differ across received signals, input-dependent fusion provides a flexible alternative to assigning fixed contributions to all scales [36,37]. Under low-SNR conditions or high inter-class similarity, for example, modulation-specific structures at some temporal resolutions may become less reliable than others [38]. Therefore, the remaining challenge is not simply to extract features at multiple temporal scales, but to determine how much each scale should contribute according to the characteristics of an individual received signal.

To provide such input-dependent flexibility, we introduce the Mixture-of-Experts (MoE) mechanism [39,40,41,42]. By assigning experts to different temporal resolutions and using a routing module to generate sample-dependent expert weights, MoE enables scale-specific representations to contribute adaptively rather than through a fixed fusion strategy. This provides a learnable mechanism for adapting multi-scale temporal modeling to the heterogeneous characteristics of modulation signals while avoiding the need to impose the same scale contribution on every input.

Motivated by these considerations, we propose MoEformer, a multi-scale Mixture-of-Experts Transformer for end-to-end AMR from raw I/Q sequences. MoEformer constructs multi-resolution signal views through temporal resampling and employs parallel experts to learn scale-specific representations. The resulting expert features are aligned to a common temporal resolution through frequency-domain resampling and adaptively fused using an input-dependent gating network that generates sample-specific expert weights. The fused representation is then processed by a Transformer encoder with Rotary Position Embedding (RoPE) [43], followed by attention pooling for sequence-level aggregation. In this way, MoEformer enables multi-scale temporal information to be modeled and combined according to the characteristics of individual received signals.

The main contributions of this work are summarized as follows:

1. We propose MoEformer, an input-adaptive multi-scale Mixture-of-Experts Transformer framework for AMR. Different temporal resolutions are modeled by scale-specific experts, and an input-dependent gating mechanism dynamically adjusts the contribution of each expert according to the characteristics of individual received signals.
2. We introduce a frequency-domain feature alignment strategy for cross-scale representation fusion. By performing spectral alignment on learned feature maps, the proposed method aligns heterogeneous temporal representations from different experts to a common temporal resolution, enabling effective multi-scale feature aggregation.
3. Experiments on three RadioML benchmarks demonstrate modest overall accuracy improvements, with more pronounced gains in the −20 to 0 dB SNR range. Ablation and complexity analyses support the key design choices while highlighting the accuracy–efficiency trade-off.

The remainder of this paper is organized as follows. Section 2 introduces the signal observation model, modeling requirements, and learning objective. Section 3 presents the proposed MoEformer framework. Section 4 reports the experimental results, including performance comparisons, computational complexity analysis, and ablation studies. Finally, Section 5 concludes the paper and discusses limitations and future work.

## 2. Problem Formulation

### 2.1. Non-Cooperative Signal Observation

In a non-cooperative spectrum-monitoring scenario, the receiver is required to identify the modulation format of an intercepted signal from a finite observation window of complex baseband samples. Let $s(t;M_{k})$ denote a transmitted waveform associated with modulation class $M_{k}$, where $M_{k}\in M=M_{1},\ldots ,M_{K}$. No instantaneous channel-state information, received signal-to-noise ratio (SNR), or residual synchronization-offset information is assumed to be available to the classifier.

A generic received signal subject to multipath propagation, carrier-frequency mismatch, phase uncertainty, and additive noise is sampled as

(1) $$r\left[n\right]={\left.\left[\left(h\left(t\right)*s\left(t;M_{k}\right)\right)e^{j(2\pi \Delta ft+\phi )}+w\left(t\right)\right]\right|}_{t=t_{n}},$$

where h(t) denotes the effective channel impulse response, ∗ denotes convolution, Δf is the carrier-frequency offset, ϕ is the residual phase offset, and w(t) represents complex additive noise.

Sampling-clock mismatch is represented through the sampling instant

(2) $$t_{n}=nT_{s}+\delta t_{n},$$

where $T_{s}$ denotes the nominal sampling period and $\delta t_{n}$ represents the timing deviation associated with sampling-clock mismatch.

The resulting finite observation is represented as

(3) $$X=\left[\begin{array}{cccc} r_{I}\left[0\right] & r_{I}\left[1\right] & \cdots & r_{I}\left[N-1\right]\\ r_{Q}\left[0\right] & r_{Q}\left[1\right] & \cdots & r_{Q}\left[N-1\right] \end{array}\right]$$

where $r_{I}\left[n\right]$ and $r_{Q}\left[n\right]$ denote the in-phase and quadrature components of r[n], respectively, and *N* denotes the length of the sequence.

### 2.2. Problem Characteristics and Modeling Requirements

Modulation-discriminative information in received I/Q sequences may occur over different temporal extents, ranging from local amplitude and phase variations to longer-range temporal dependencies. The relative informativeness of these temporal structures may also vary with modulation type, SNR, and channel impairments. Consequently, a representation constructed at a single temporal resolution cannot be assumed to provide equally effective discrimination for all received signals.

Multi-scale representations provide complementary temporal views but raise two key requirements: representations extracted at different temporal scales must be mapped to a common resolution to enable consistent feature aggregation, and the relative contributions of different scales should be determined according to the characteristics of each input rather than by fixed fusion weights. Therefore, effective multi-scale AMR requires both cross-scale feature alignment and input-dependent fusion.

### 2.3. Learning Objective and Scope

Given an observation X, AMR is formulated as a supervised closed-set *K*-class classification problem. The model learns a parameterized mapping $f_{\theta }\left(\cdot \right)$ and predicts

(4) $$\hat{y}=\arg\max_{M_{k}\in M}P_{\theta }\left(M_{k}|X\right).$$

For a training set $D={\left\{\left(X_{m},y_{m}\right)\right\}}_{m=1}^{N_{D}}$ containing observations across multiple SNR and impairment conditions, the model parameters are optimized using the cross-entropy objective

(5) $$\theta^{*}=\arg\min_{\theta }-\frac{1}{N_{D}}\sum_{m=1}^{N_{D}}\log P_{\theta }\left(y_{m}|X_{m}\right).$$

We perform closed-set AMR directly from finite complex-baseband I/Q observations without providing channel state, SNR, or synchronization offsets as auxiliary inputs, and no explicit channel estimation, equalization, or synchronization compensation is applied. The resulting task is therefore to learn scale-specific representations and adaptively integrate them under unknown signal impairments.

## 3. Method

**Figure 1:**
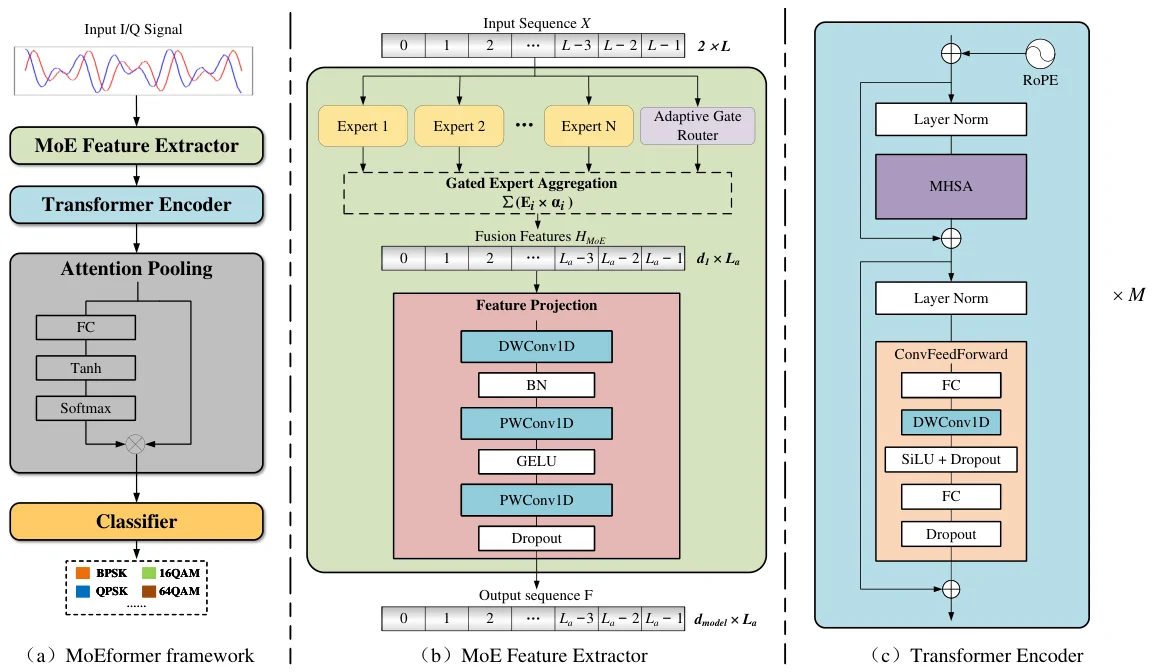
Overall architecture of the proposed MoEformer.

As illustrated in Figure 1, we propose MoEformer, an adaptive multi-scale mixture-of-experts framework for automatic modulation recognition. The proposed framework consists of three sequential stages. First, the MoE feature extractor generates multiple temporally resampled signal views and extracts scale-specific local features through parallel expert branches. Since temporal resampling results in expert features with different sequence lengths, a frequency-domain feature alignment operation maps them to a common temporal resolution, after which an input-dependent gating mechanism adaptively fuses the multi-scale representations. Second, the fused features are processed by stacked Transformer encoders equipped with RoPE to capture long-range temporal dependencies. Finally, attention-based temporal pooling aggregates the encoded sequence into a compact sequence-level representation for modulation classification.

### 3.1. MoE Feature Extractor

#### 3.1.1. Time-Domain Resampling

Let $X\in R^{B\times 2\times L}$ denote a batch of normalized I/Q sequences, where *B* is the batch size and *L* is the input sequence length. To expose the feature extractor to temporal structures at different resolutions, we construct *N* resampled views of each input using a predefined set of scale factors ${\left\{s_{i}\right\}}_{i=1}^{N}$. The input to the *i*-th expert is given by

(6) $$X_{i}=T_{s_{i}}\left(X\right)\in R^{B\times 2\times L_{i}},\;L_{i}=\left\lfloor s_{i}L\right\rfloor ,$$

where $T_{s_{i}}\left(\cdot \right)$ denotes one-dimensional linear interpolation along the temporal axis.

**Figure 2:**
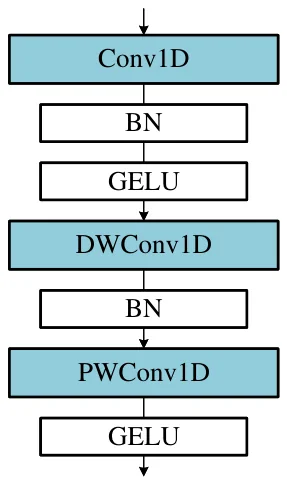
Architecture of the Local Representation Block (LRB).

Each resampled sequence is subsequently processed by an independent Local Representation Block (LRB). All expert branches share the same architectural configuration but maintain separate learnable parameters, allowing individual experts to learn scale-specific representations from their corresponding temporal resolutions. As illustrated in Figure 2, the output of the *i*-th expert is formulated as

(7) $$H_{i}={\mathrm{LRB}}_{i}\left(X_{i}\right)\in R^{B\times C_{e}\times \ell_{i}},$$

where $C_{e}$ denotes the number of output channels and $\ell_{i}$ denotes the temporal length of the resulting feature map.

The LRB first employs a standard 1D convolution to extract local temporal patterns from the resampled I/Q sequence. A depthwise convolution is then applied to further model local temporal dependencies with limited computational overhead, followed by a pointwise convolution [44] for channel-wise feature interaction and projection. Batch normalization [45] and GELU activation [46] are incorporated within the block, and dropout is applied after the final projection for regularization. This lightweight design provides a common architectural template across temporal scales while allowing independently parameterized experts to learn scale-dependent representations.

#### 3.1.2. Frequency-Domain Feature Alignment

**Figure 3:**
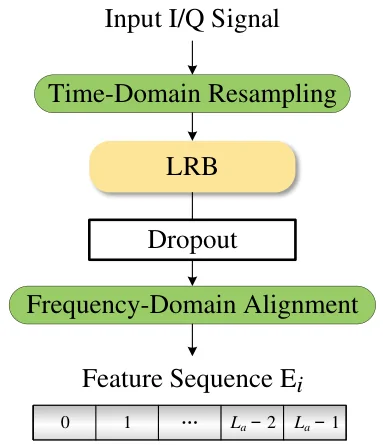
Processing pipeline of a scale-specific expert.

As depicted in the final stage of Figure 3, the multi-scale intermediate features extracted by the LRB have different temporal lengths due to temporal resampling, making direct fusion infeasible. To enable consistent multi-scale fusion, we align the expert features to a predefined target temporal length $L_{a}$ in the frequency domain.

Specifically, for the *i*-th expert feature $H_{i}$, we first apply a real Fast Fourier Transform (rFFT) along the temporal dimension. The resulting one-sided spectrum is then adjusted to the target spectral length through a spectral length-matching operator $P(\cdot ,L_{a})$, followed by an inverse rFFT to reconstruct the aligned time-domain feature $E_{i}$. The alignment process is formulated as

(8) $$\begin{array}{cc} S_{i} & =F_{\ell_{i}}\left(H_{i}\right)\in C^{B\times C_{e}\times \left(\left\lfloor \ell_{i}/2\right\rfloor +1\right)},\\ {\tilde{S}}_{i} & =\sqrt{\frac{L_{a}}{\ell_{i}}}\;P_{\ell_{i}\to L_{a}}\left(S_{i}\right)\in C^{B\times C_{e}\times \left(\left\lfloor L_{a}/2\right\rfloor +1\right)},\\ E_{i} & =F_{L_{a}}^{-1}\left({\tilde{S}}_{i}\right)\in R^{B\times C_{e}\times L_{a}}, \end{array}$$

where F(·) and $F^{-1}\left(\cdot \right)$ denote the orthonormally normalized temporal rFFT and inverse rFFT, respectively. Since $H_{i}$ is real-valued, only the $\lfloor \ell_{i}/2\rfloor +1$ non-negative frequency components are represented. The spectral length-matching operator $P(\cdot ,L_{a})$ maps the one-sided spectrum to $\lfloor L_{a}/2\rfloor +1$ components by preserving the overlapping low-frequency components and padding or truncating the remaining high-frequency components as required. For even-length sequences, the Nyquist component is handled consistently with the one-sided real-valued Fourier representation. The aligned feature $E_{i}$ is then reconstructed by the inverse rFFT with output length $L_{a}$.

It should be noted that the Fourier transform is performed on the learned temporal feature maps rather than directly on the raw I/Q signal. The frequency-domain alignment therefore provides a consistent mechanism for mapping heterogeneous latent representations to a common temporal resolution while retaining the dominant low-frequency structure of the learned features. This unified representation facilitates subsequent cross-scale feature fusion.

#### 3.1.3. Adaptive Gating and Feature Projection

A limitation of static multi-scale fusion is that it assigns fixed contributions to different temporal scales regardless of the input. However, the relative importance of these scales may vary across modulation types and channel conditions. To address this issue, we introduce an input-dependent gating mechanism to adaptively determine the contribution of each expert.

**Figure 4:**
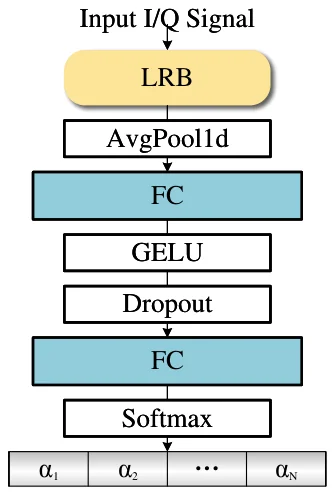
Input-adaptive expert gating.

As illustrated in the routing branch of Figure 4, the gating network first processes the input sequence X using the same basic architectural pattern as the expert LRBs, while allowing branch-specific hyperparameters such as the convolution kernel size and channel width. The resulting feature map is then aggregated into a global routing vector through temporal global average pooling:

(9) $$g=\mathrm{GAP}\left(\mathrm{LRB}(X)\right)\in R^{B\times C_{g}},$$

where LRB(·) denotes the lightweight gating encoder, $C_{g}$ denotes its output channel dimension, and GAP(·) denotes global average pooling along the temporal dimension.

The normalized routing weights are subsequently generated by a multi-layer perceptron (MLP) followed by a Softmax activation:

(10) $$\alpha =\mathrm{Softmax}\left(\mathrm{MLP}(g)\right)\in R^{B\times N},$$

where $\sum_{i=1}^{N}\alpha_{b,i}=1$ for each input instance *b*. Unlike sparse MoE routing, we adopt a dense soft-gating strategy in which all experts participate in feature aggregation with input-dependent weights. This allows complementary temporal information captured at different resolutions to contribute jointly to the fused representation.

The fused multi-scale representation is obtained by the weighted aggregation of the aligned expert features:

(11) $$H_{\mathrm{MoE}}=\sum_{i=1}^{N}E_{i}.\alpha_{i}$$

For notational simplicity, $\alpha_{i}$ denotes the batch-wise routing weight associated with the *i*-th expert, which is broadcast along the channel and temporal dimensions during feature aggregation.

Finally, the fused representation is further refined and projected to the embedding dimension required by the subsequent Transformer encoder. As illustrated in the Feature Projection block of Figure 1b, the projection is formulated as

(12) $$F=P_{\mathrm{proj}}\left(H_{\mathrm{MoE}}\right)\in R^{B\times d_{\mathrm{model}}\times L_{a}},$$

where $P_{\mathrm{proj}}\left(\cdot \right)$ consists of a DWConv1D with Batch Normalization, followed by a PWConv1D with GELU activation and a final PWConv1D with Dropout. This projection refines the fused local representation and maps its channel dimension to $d_{\mathrm{model}}$ for subsequent global temporal modeling.

### 3.2. Transformer-Based Global Temporal Modeling

While the MoE front-end extracts scale-specific local features, AMR also requires modeling long-range temporal dependencies. Channel-induced temporal correlations, phase variations, and symbol timing relationships may not be sufficiently captured by localized convolutional operations alone. Therefore, we employ a Transformer encoder stack to further model global temporal dependencies.

Following the feature projection module, the feature map is transposed to form the input sequence $F\in R^{B\times L_{a}\times d_{\mathrm{model}}}$, where $L_{a}$ denotes the aligned temporal length and $d_{\mathrm{model}}$ is the embedding dimension. Corresponding to Figure 1c, the sequence is subsequently processed by a stack of *M* pre-normalized Transformer encoder blocks, where M=2 in all experiments. Each block consists of a Multi-Head Self-Attention (MHSA) module equipped with RoPE, followed by a Convolutional Feed-Forward Network (CFFN).

#### 3.2.1. Multi-Head Self-Attention with RoPE

For the *l*-th Transformer block, the input feature $F_{l}\in R^{B\times L_{a}\times d_{\mathrm{model}}}$ is first normalized and linearly projected into query (Q), key (K), and value (V) representations:

(13) $$Q,K,V=W_{Q,K,V}\left(\mathrm{LN}(F_{l})\right),$$

where each representation has an embedding dimension of $d_{\mathrm{model}}$. The representations are then divided into *H* attention heads, each with a dimensionality of $d_{h}=d_{\mathrm{model}}/H$.

Absolute positional encoding associates positional information with fixed temporal indices, whereas modulation-related temporal patterns are often characterized by relative relationships among different temporal positions. To introduce relative positional information into the attention mechanism, we apply RoPE to the queries and keys. For a feature pair $\left(x_{2k},x_{2k+1}\right)$ at temporal position *t*, the rotary transformation is defined as

(14) $$\left(\begin{array}{c} x_{2k}^{\prime }\\ x_{2k+1}^{\prime } \end{array}\right)=\left(\begin{array}{cc} \cos\theta_{t} & -\sin\theta_{t}\\ \sin\theta_{t} & \cos\theta_{t} \end{array}\right)\left(\begin{array}{c} x_{2k}\\ x_{2k+1} \end{array}\right),$$

Here, $\theta_{t}$ is a shorthand for the pair-specific rotary angle $\theta_{t,k}=t\omega_{k}$, where $\omega_{k}$ denotes the rotary frequency associated with the *k*-th feature pair. The rotation is applied independently within each attention head, introducing relative positional information into the learned embedding space for temporal dependency modeling.

Let $\tilde{Q}$ and $\tilde{K}$ denote the rotated queries and keys. The MHSA module captures global dependencies through scaled dot-product attention. Incorporating the residual connection, the output of the attention module is formulated as

(15) $$Z_{l}=F_{l}+\mathrm{MHSA}\left(\tilde{Q},\tilde{K},V\right),$$

where MHSA(·) performs scaled dot-product attention independently across all *H* heads, concatenates the resulting head outputs, and applies a linear output projection.

#### 3.2.2. Convolutional Feed-Forward Network (CFFN)

To complement the global attention mechanism with local temporal refinement, we replace the standard position-wise FFN with a Convolutional Feed-Forward Network. The complete forward pass for the remaining part of the *l*-th block is given by

(16) $$\begin{array}{cc} \mathrm{CFFN}(Z_{l}) & =L_{2}\;\left(\sigma \;\left(\mathrm{DWConv}1D(L_{1}\left(Z_{l}\right))\right)\right),\\ F_{l+1} & =Z_{l}+\mathrm{CFFN}\left(\mathrm{LN}\left(Z_{l}\right)\right), \end{array}$$

where $L_{1}$ and $L_{2}$ denote linear projections for channel reduction and restoration, respectively, DWConv1D(·) performs depthwise temporal convolution, and σ(·) denotes the SiLU activation. Dropout is applied within the CFFN for regularization. The depthwise temporal convolution introduces local interactions into the otherwise position-wise feed-forward transformation, complementing the global dependencies captured by the self-attention module.

### 3.3. Attention-Based Temporal Aggregation and Classification

The output of the Transformer encoder remains a temporal sequence, whereas AMR requires a sequence-level representation for final classification. Uniform temporal aggregation assigns equal importance to all temporal positions and may therefore weaken discriminative cues when their contributions vary across the observation window. To address this issue, we employ an attention-based pooling mechanism to adaptively aggregate the encoded temporal features.

Let $Z=\left[z_{1},z_{2},\ldots ,z_{L_{a}}\right]\in R^{B\times L_{a}\times d_{\mathrm{model}}}$ denote the output sequence of the final Transformer encoder. For each temporal position *t*, a scalar attention score is computed as

(17) $$e_{t}=\tanh\left(w^{⊤}z_{t}+b\right),$$

where $w\in R^{d_{\mathrm{model}}}$ and *b* are learnable parameters. The normalized attention weight is then obtained by applying a Softmax operation along the temporal dimension:

(18) $$\beta_{t}=\frac{\exp(e_{t})}{\sum_{j=1}^{L_{a}}\exp\left(e_{j}\right)}.$$

The sequence-level representation is computed as the weighted sum of the temporal features:

(19) $$z=\sum_{t=1}^{L_{a}}\beta_{t}z_{t},\;\sum_{t=1}^{L_{a}}\beta_{t}=1.$$

Compared with average pooling, this data-dependent aggregation strategy assigns different contributions to different temporal positions, allowing informative intervals to receive larger weights while reducing the influence of less discriminative or noise-dominated regions. The aggregated representation z is finally fed into a linear classifier to produce the modulation-class logits, from which the corresponding class probabilities are obtained through a Softmax operation.

## 4. Experiments and Results

### 4.1. Evaluation Datasets and Experimental Setup

As summarized in Table 1, we evaluate MoEformer on three widely used AMR benchmarks: RadioML2016.10a [11], RadioML2016.10b [16], and RadioML2018.01A [14]. RadioML2016.10a contains 220,000 2×128 I/Q samples from 11 modulation classes under 20 SNR levels (−20 to 18 dB). RadioML2016.10b contains 1.2 million 2×128 I/Q samples from 10 modulation classes with the same SNR range. RadioML2018.01A provides 2,555,904 2×1024 I/Q samples covering 24 modulation classes and 26 SNR levels (−20 to 30 dB).

Beyond the differences in modulation categories, sequence length and dataset magnitude, all three benchmarks simulate realistic non-ideal wireless reception environments by incorporating practical channel and hardware impairments. Specifically, RadioML2016.10a and RadioML2016.10b are constructed via the GNU Radio dynamic channel model, considering additive white Gaussian noise (AWGN), center-frequency offset (CFO), sample-rate offset (SRO), and multipath fading [11,16]. By contrast, RadioML2018.01A characterizes complex non-ideal propagation scenarios involving AWGN, carrier-frequency offset, multipath fading, and delay spread [14].

For all datasets, samples are split into training, validation, and test sets with a ratio of 6:2:2 within each modulation-SNR pair. All model configurations and hyperparameters, including the temporal scale factors of the expert branches, were selected exclusively according to validation-set performance. The test set was used only for the final performance evaluation. Prior to training, all I/Q sequences were L2-normalized. All experiments were implemented in PyTorch 2.4.0 (compiled with CUDA 12.1) and conducted on a single NVIDIA RTX 4090 GPU. The model was trained using cross-entropy loss and optimized by AdamW with an initial learning rate of 0.001 and a weight decay coefficient of 0.01. The learning rate was scheduled by ReduceLROnPlateau, where the learning rate is multiplied by 0.5 once the validation loss fails to improve for five consecutive epochs. The maximum number of training epochs is set to 100, and early stopping with a patience of 15 epochs is adopted to prevent overfitting. The batch size is set to 128 for RadioML2016.10a and RadioML2016.10b, and 512 for RadioML2018.01A.

Table 2 summarizes the base configuration used for RadioML2016.10a and RadioML2016.10b. For RadioML2018.01A, we retain the overall MoEformer topology while slightly increasing the capacity of the local expert extractor to accommodate the longer observation window and substantially broader modulation space. Specifically, each expert is configured as Conv1D (2→20), DWConv1D (20→20), PWConv1D (20→40), DWConv1D (40→40), and PWConv1D (40→60). The standard and depthwise convolutions use a temporal kernel size of 15, whereas all pointwise convolutions use a kernel size of 1. Accordingly, the feature-projection module takes the resulting 60-channel expert representation as input while preserving the Transformer embedding dimension at $d_{\mathrm{model}}=128$. The CFFN bottleneck dimension is also increased from 32 to 48. All other components remain unchanged across the three datasets.

### 4.2. Overall Performance Comparison

We compare MoEformer with six representative deep-learning-based AMR baselines, including ResNet [14], AMC-Net [47], FEA-T [48], MCLDNN [24], MCformer [35], and TLDNN [49]. ResNet, AMC-Net, FEA-T, MCLDNN, and MCformer operate directly on raw I/Q sequences, whereas TLDNN uses amplitude–phase representations transformed from the original I/Q samples.

**Figure 5:**
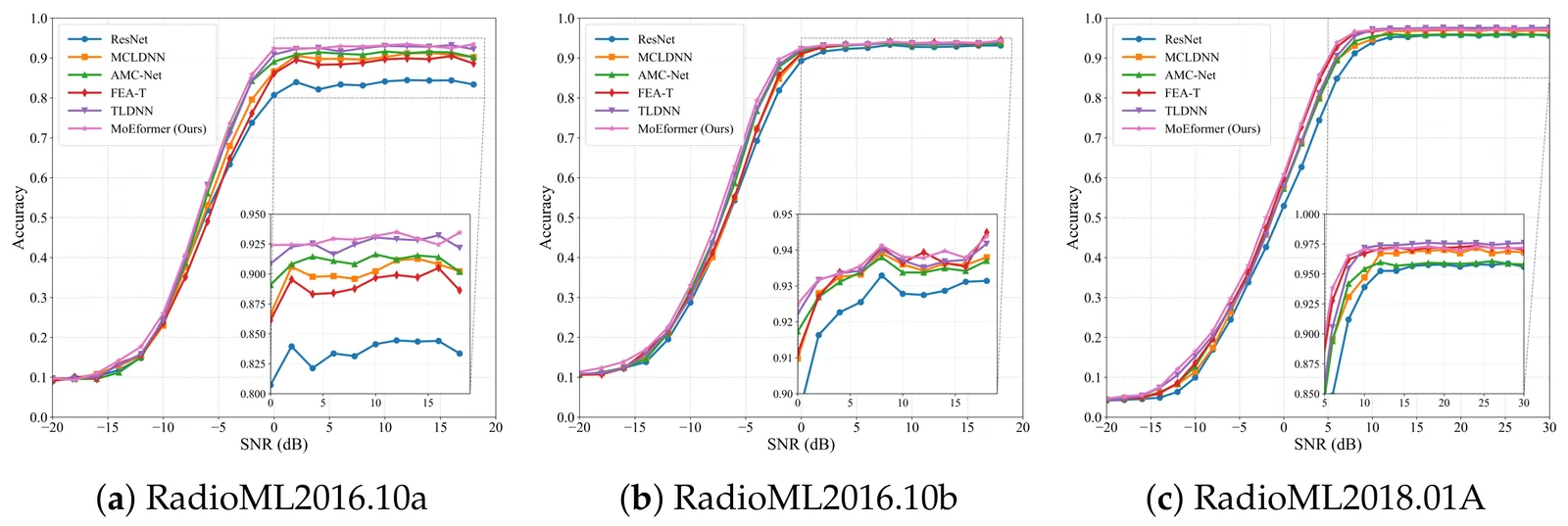
Comparison of classification accuracy versus SNR across three benchmark datasets.

The overall accuracy is calculated over the entire test set, including all modulation classes and SNR levels. As reported in Table 3 and Figure 5, MoEformer achieves the highest overall average classification accuracy on all three benchmark datasets, reaching 63.74%, 66.24%, and 64.22% on RadioML2016.10a, RadioML2016.10b, and RadioML2018.01A, respectively. Compared with the best-performing baseline on each dataset, the corresponding improvements are 0.74 percentage points over TLDNN on RadioML2016.10a, 0.91 percentage points over TLDNN on RadioML2016.10b, and 0.85 percentage points over FEA-T on RadioML2018.01A. Although these margins are relatively modest, the results show that MoEformer maintains consistently competitive aggregate performance across datasets with different sequence lengths and modulation configurations.

In the SNR range from −20 to 0 dB, MoEformer achieves average accuracies of 39.87%, 43.72%, and 22.87% on the three datasets, respectively, exceeding the strongest baseline in this range by 1.07, 1.56, and 1.43 percentage points. For SNRs >0 dB, the performance differences become considerably smaller. MoEformer achieves accuracies of 92.92%, 93.77%, and 94.55%, with improvements of only 0.35 and 0.12 percentage points over the strongest baseline on RadioML2016.10a and RadioML2016.10b, respectively, while achieving an accuracy 0.22 percentage points lower than FEA-T on RadioML2018.01A. These results suggest that the benefits of adaptive fusion across multiple temporal scales are more evident under noisy conditions, whereas the recognition performance of different methods becomes increasingly comparable at higher SNRs. Therefore, the main advantage of MoEformer lies in its improved recognition performance under challenging signal conditions rather than consistently large improvements across all SNR levels. The tradeoff between recognition accuracy and computational efficiency is further discussed in Section 4.3.

**Figure 6:**
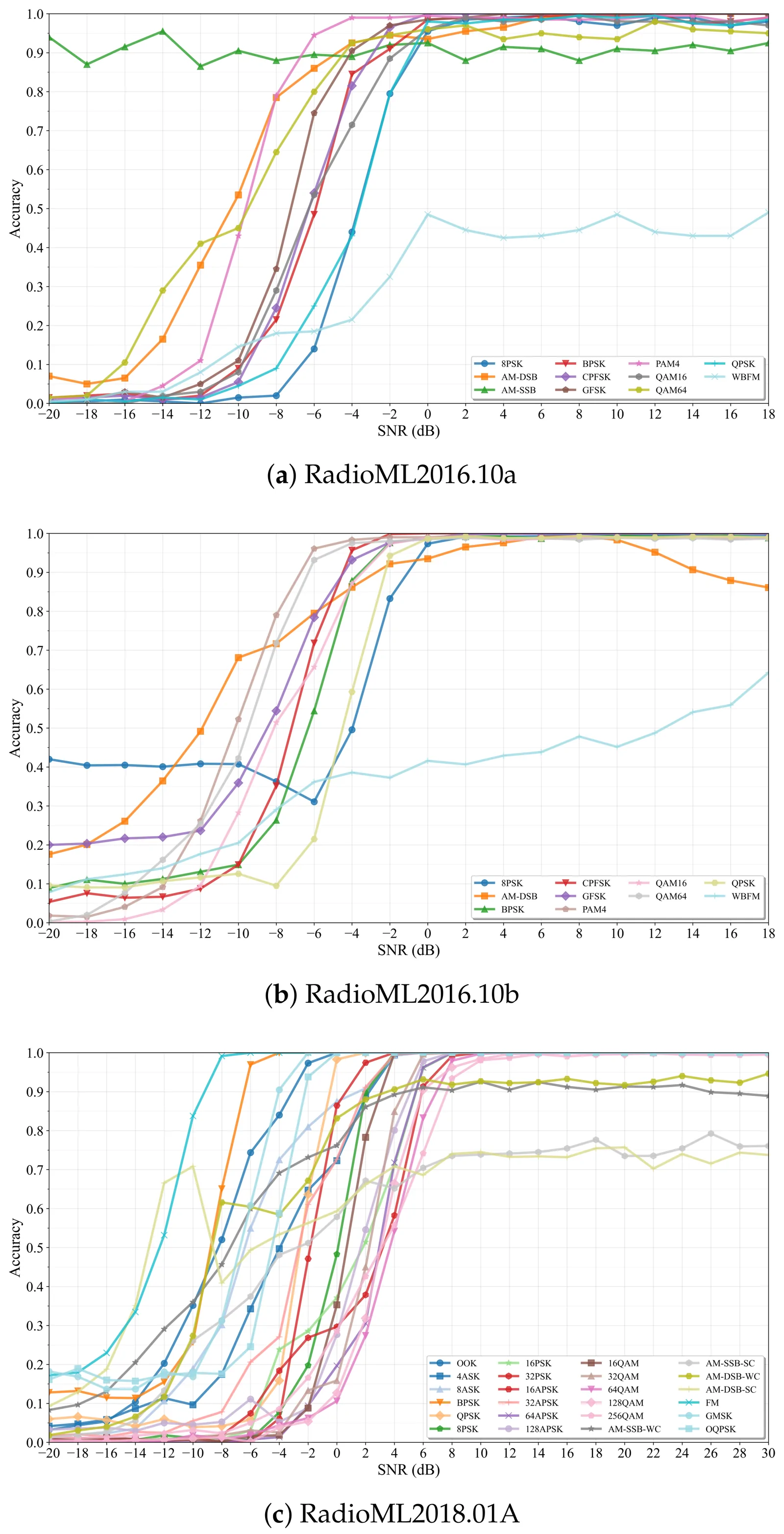
Performance of different modulation schemes on three benchmark datasets.

**Figure 7:**
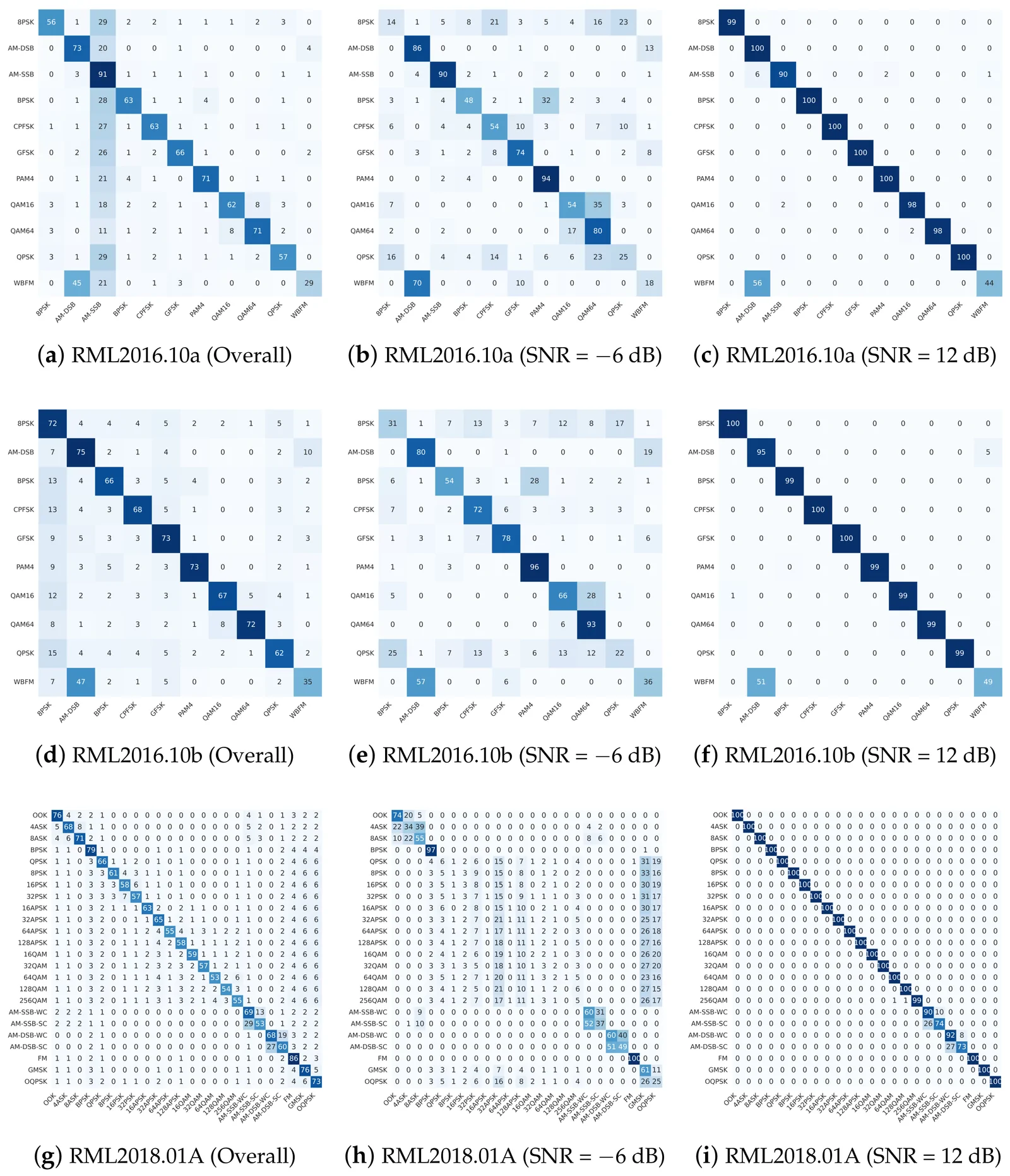
Confusion matrices for modulation classification across three benchmark RadioML datasets under varying SNR conditions.

The per-class SNR curves in Figure 6 further show that recognition difficulty varies substantially across modulation types. On RadioML2016.10a and RadioML2016.10b, WBFM and AM-DSB remain among the more difficult classes, with WBFM showing comparatively slow performance improvement even as the SNR increases. This difficulty inherently stems from the dataset generation process, where silent periods in the source audio cause these signals to temporarily degrade into indistinguishable unmodulated carriers. Most digital modulation schemes, in contrast, approach relatively high recognition accuracy once the SNR becomes positive. On RadioML2018.01A, several high-order QAM, PSK, APSK, and amplitude-modulated variants exhibit slower convergence or lower saturation accuracy than the easier classes. This behavior is consistent with the greater similarity among closely related modulation formats and their sensitivity to channel impairments. As illustrated in Figure 7, the confusion matrices provide a more detailed view of the classification errors under different SNR conditions. On RadioML2016.10a and RadioML2016.10b, the errors are relatively dispersed at −6 dB, whereas most digital modulation classes become clearly distinguishable at 12 dB. A notable exception is the persistent confusion between WBFM and AM-DSB. Even at 12 dB, a substantial fraction of WBFM samples are still classified as AM-DSB, leaving the recognition accuracy of WBFM below 50%. This observation suggests that increasing SNR substantially reduces broad noise-induced errors, but does not completely resolve the ambiguity between these two analog modulation classes.

For RadioML2018.01A, the confusion matrices exhibit a similar SNR-dependent transition, although the larger number of modulation classes results in more diverse error patterns. At −6 dB, misclassifications are distributed across multiple related modulation families. As the SNR increases to 12 dB, most classes show substantially improved separability, while the remaining errors become concentrated in a smaller number of closely related categories, including several amplitude-modulated variants and high-order digital modulation schemes. In particular, residual confusion can still be observed among structurally similar AM-SSB/AM-DSB variants and among some higher-order PSK, APSK, and QAM categories. Overall, the evolution of the confusion matrices is consistent with the SNR-dependent trends observed in Figure 6: increasing SNR reduces widespread classification errors, whereas classes with closely related signal characteristics remain comparatively difficult to separate.

### 4.3. Complexity and Efficiency Analysis

In addition to recognition accuracy, the computational characteristics of an AMR model are important for practical deployment. Table 3 therefore compares all evaluated methods in terms of parameter count, floating-point operations (FLOPs), and inference time.

In terms of model size, MoEformer maintains a moderate parameter count while achieving the highest overall average accuracy on all three datasets. It contains approximately 187.7K parameters on RadioML2016.10a/b and 237.3K on RadioML2018.01A, considerably fewer than AMC-Net, MCLDNN, and TLDNN, although larger than ResNet, FEA-T, and MCformer. Thus, its accuracy improvements do not rely on a large model capacity.

In terms of computational cost, MoEformer requires more FLOPs than several lightweight baselines on the shorter-sequence datasets, while remaining substantially less expensive than AMC-Net and MCLDNN. On the longer RadioML2018.01A sequences, MoEformer remains computationally competitive with several baselines, suggesting that its additional architectural complexity does not lead to a disproportionate computational burden under the evaluated long-sequence setting. Its inference times are 1.6955, 1.6901, and 1.8196 ms, respectively. The higher latency is plausibly attributable to the execution of multiple expert branches, whose theoretical parallelism may not translate into fully overlapping GPU execution.

Overall, the results in Table 3 indicate a trade-off between recognition accuracy and computational efficiency. MoEformer achieves modest improvements in overall accuracy, with more noticeable gains in the −20 to 0 dB SNR range, but requires longer inference times than several lightweight baselines. The single-expert ablation results, presented in Section 4.6.1, further suggest that the multi-expert design contributes to the additional inference overhead. These findings suggest that MoEformer may be considered for accuracy-oriented AMR applications where inference latency is less critical, while further optimization is needed to improve its computational efficiency.

### 4.4. Visualization and Representation Analysis

**Figure 8:**
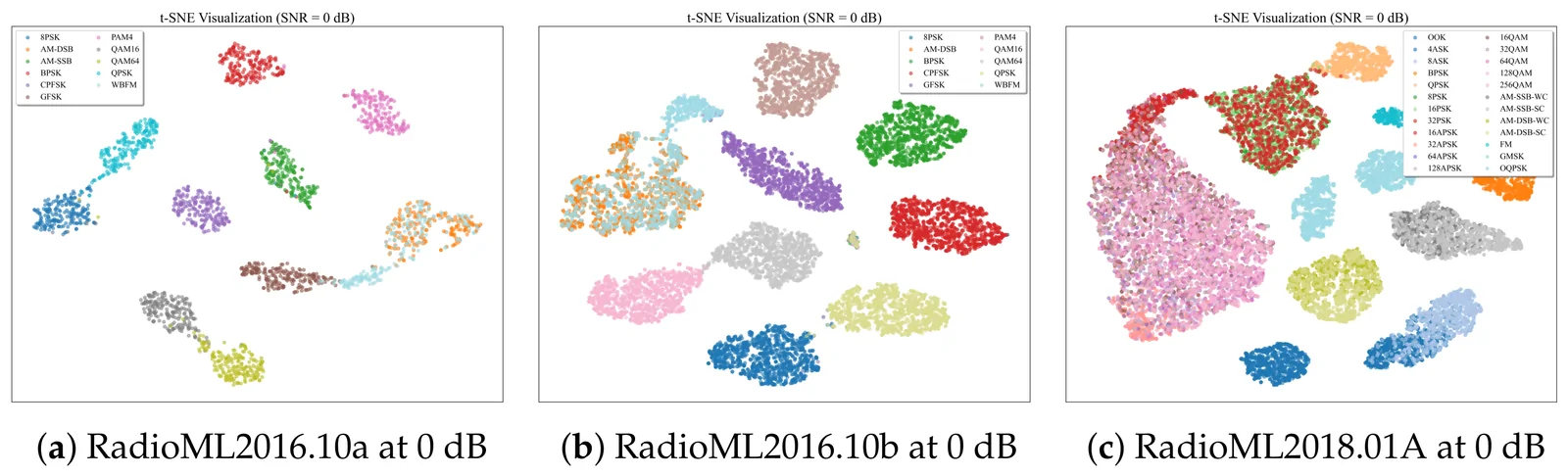

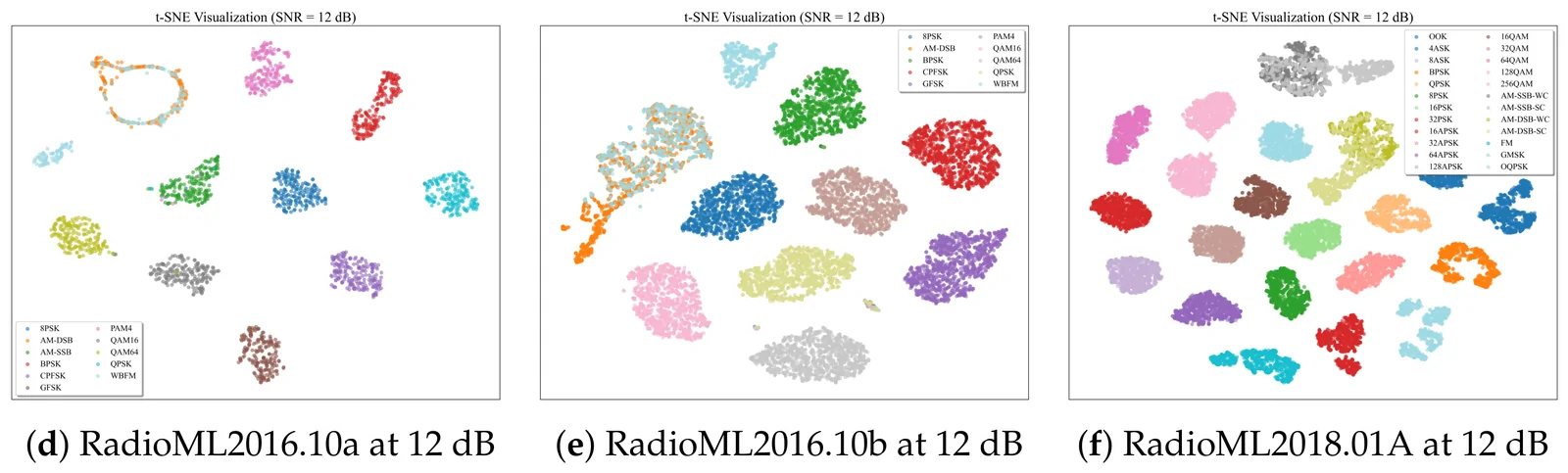
t-SNE visualizations of learned modulation feature representations across three benchmark datasets at 0 dB and 12 dB SNR.

As shown in Figure 8, t-SNE [50] is employed to qualitatively visualize the learned feature representations of the proposed method on RadioML2016.10a, RadioML2016.10b, and RadioML2018.01A at SNRs of 0 and 12 dB. At 0 dB, several modulation classes in the RadioML2016 datasets already exhibit discernible local clustering patterns, although varying degrees of inter-class mixing remain. In particular, the representations of AM-DSB and WBFM show substantial overlap, while some visually adjacent regions can also be observed for modulation classes with relatively similar signal characteristics. The embedding of RadioML2018.01A appears more complex at 0 dB, with several closely related modulation families exhibiting considerable local intermixing, reflecting the increased difficulty associated with the larger number of modulation categories under noisy conditions. At 12 dB, the embeddings of many modulation classes become visually more compact and distinguishable, suggesting that the learned representation benefits from the increased availability of modulation-discriminative information at higher SNRs. Nevertheless, the degree of separation is not uniform across modulation types. In particular, AM-DSB and WBFM remain partially intermixed in the RadioML2016 datasets, while several closely related classes in RadioML2018.01A still exhibit local proximity, irregular cluster shapes, or limited overlap. Overall, these visualizations provide qualitative evidence that the learned feature space tends to exhibit improved class organization as the SNR increases, while also highlighting the persistent difficulty of separating modulation types with highly similar signal characteristics.

### 4.5. Sensitivity Analysis of Multi-Scale Configuration

Table 4 investigates the impact of expert number and temporal scale diversity on recognition performance. The results show that different expert configurations exhibit varying recognition performance across the three datasets. Increasing the number of experts from two to four generally improves performance when complementary temporal resolutions are introduced. However, further increasing the number of experts to five does not provide additional accuracy gains, despite increasing computational cost.

The performance of different temporal scale combinations varies across datasets. The configuration [0.5,1.0,1.5,2.0] achieves better performance on RadioML2016.10a and RadioML2018.01A, while [0.5,0.75,1.0,2.0] performs better on RadioML2016.10b. These results suggest that the effectiveness of multi-scale modeling depends on the diversity and complementarity of temporal resolutions rather than simply increasing the number of experts.

Considering recognition performance and computational cost, the four-expert configuration provides a favorable balance between representation diversity and model complexity and is therefore adopted in the final model.

### 4.6. Ablation Study of MoEformer Components

We conduct ablation experiments to quantify the individual contributions of MoEformer’s core designs. Specifically, we investigate the multi-scale expert mechanism together with adaptive routing and cross-scale feature alignment, and finally positional encoding alongside temporal aggregation strategies.

#### 4.6.1. Effect of Multi-Scale Expert Modeling and Adaptive Gating Mechanism

Table 5 evaluates the contributions of the three key components in the proposed MoE front-end: multi-expert representation learning, input-adaptive routing, and frequency-domain feature alignment. Specifically, *Single Expert (w/o MoE)* removes the multi-scale expert structure and routing mechanism; *Uniform Expert Weighting* replaces the adaptive routing weights with fixed equal weights ($\alpha_{i}=1/N$); and *Time-Domain Alignment* substitutes the proposed frequency-domain alignment with direct temporal interpolation.

Removing the multi-expert structure leads to accuracy decreases on all three datasets, with reductions of 0.70, 0.71, and 2.60 percentage points on RadioML2016.10a, RadioML2016.10b, and RadioML2018.01A, respectively. The larger degradation on RadioML2018.01A indicates that multi-scale expert representations become more beneficial when modeling longer sequences and more diverse modulation categories.

Replacing the input-dependent routing weights with uniform expert fusion consistently reduces recognition performance, decreasing the overall accuracy to 63.27%, 65.57%, and 62.68% on the three datasets. This verifies that adaptive expert weighting provides advantages over fixed multi-scale fusion, particularly under challenging signal conditions.

Furthermore, replacing frequency-domain feature alignment with time-domain interpolation results in performance degradation across all datasets. This demonstrates that the proposed alignment strategy provides a more consistent solution for integrating multi-scale expert representations.

#### 4.6.2. Effect of Temporal Aggregation and Relative Positional Encoding

**Figure 9:**
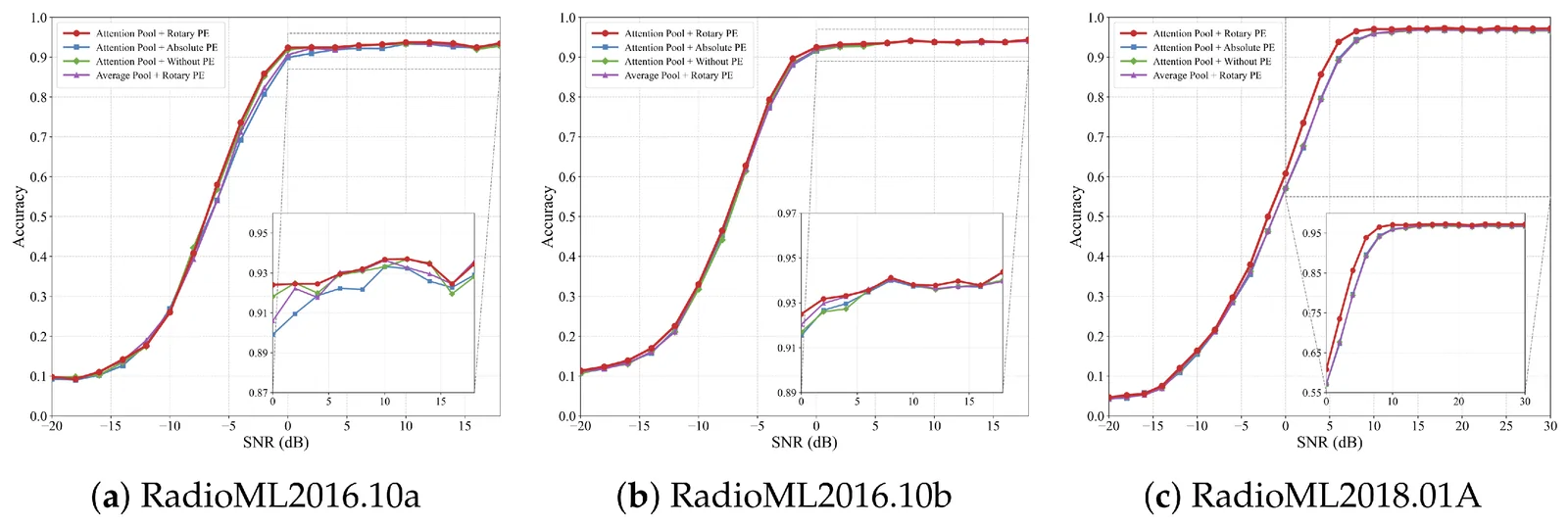
Classification accuracy versus SNR for different temporal aggregation and positional encoding configurations.

We further evaluate the effects of temporal aggregation and positional encoding while keeping the remaining model configuration unchanged. As shown in Figure 9, the effect of temporal aggregation is examined by comparing attention pooling with average pooling under the same RoPE setting, while the effect of positional encoding is evaluated by comparing RoPE, absolute positional encoding, and no positional encoding under attention pooling.

For temporal aggregation, attention pooling generally achieves slightly higher recognition accuracy than average pooling across the three datasets. The difference is relatively small on RadioML2016.10a and RadioML2016.10b, but becomes more noticeable on RadioML2018.01A, particularly in the SNR range where the recognition accuracy increases rapidly. This indicates that assigning different contributions to temporal positions can improve sequence-level aggregation compared with uniform averaging. The larger difference observed on RadioML2018.01A also suggests that such adaptive aggregation is more useful when longer temporal sequences need to be summarized.

For positional encoding, RoPE, absolute positional encoding, and no positional encoding show similar overall trends on RadioML2016.10a and RadioML2016.10b. RoPE and the no-PE configuration perform comparably over most SNRs, whereas absolute positional encoding is slightly less competitive at several medium-to-high SNR points. A clearer difference is observed on RadioML2018.01A, where RoPE generally achieves higher accuracy than the other two configurations over the intermediate-SNR range. The performance gap becomes smaller at high SNR as the three curves approach saturation. Since RadioML2018.01A uses a substantially longer input sequence than the two RadioML2016 datasets, this result suggests that relative positional information can provide greater benefit when modeling longer temporal contexts.

### 4.7. Routing Weight Analysis

**Figure 10:**
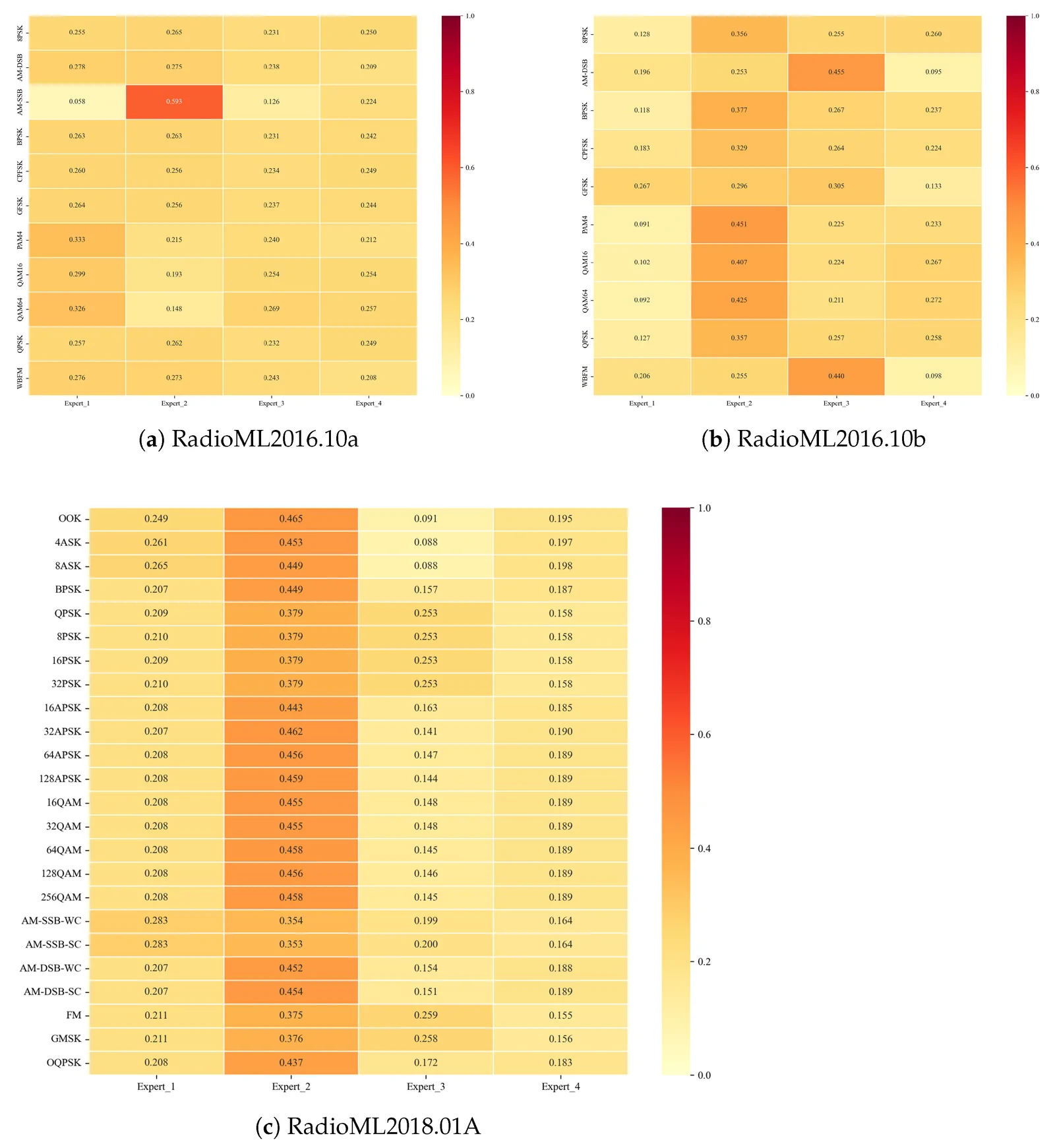
Distribution of gating weights across modulation types on the RadioML datasets.

To further examine the behavior of the adaptive gating mechanism, we analyze the average routing weights across different modulation classes, as shown in Figure 10. The routing distributions reveal that the gating network learns non-uniform expert combinations across modulation classes. Although multiple experts contribute simultaneously due to the dense soft-gating strategy, different classes exhibit distinct weight preferences, with some assigning larger contributions to specific experts. For instance, AM-SSB on RadioML2016.10a assigns a weight of 0.593 to Expert 2. Similar class-dependent routing patterns are observed on RadioML2016.10b and RadioML2018.01A, indicating that the proposed method dynamically adjusts multi-scale feature contributions according to modulation characteristics instead of relying on a fixed fusion scheme.

## 5. Conclusions

This study presented MoEformer, an end-to-end input-adaptive multi-scale Mixture-of-Experts Transformer for automatic modulation recognition from raw complex-baseband I/Q sequences. The central contribution is an adaptive mechanism that determines how much information should be obtained from different temporal resolutions for each received signal. By combining scale-specific representation learning, frequency-domain feature alignment, and learned soft routing, MoEformer replaces fixed multi-scale fusion with a more flexible representation strategy.

Experiments on three public AMR benchmarks show that MoEformer achieves modest overall improvements over the compared methods. The advantage of MoEformer is more pronounced under low-to-moderate SNR conditions, where noise and channel impairments make modulation-related features more difficult to distinguish, while its performance remains competitive at higher SNRs. This indicates that the main benefit of input-adaptive multi-scale modeling lies in improving recognition under challenging signal conditions rather than producing uniformly large gains across all operating regimes. The ablation results further support the contributions of adaptive routing and cross-scale alignment. The results indicate a trade-off between the improved recognition performance under challenging signal conditions and the additional computational overhead introduced by the multi-expert architecture.

MoEformer holds application potential for intelligent spectrum monitoring, cognitive radio sensing, non-cooperative signal analysis, and distributed or edge-based spectrum sensor networks. Because it operates directly on raw I/Q data, it can reduce dependence on manually designed features and facilitate integration with receivers operating under changing wireless conditions. In principle, the model could also serve as a modulation identification module for subsequent tasks such as signal cataloging, interference awareness, dynamic spectrum access, and wireless situational awareness. Its input-adaptive routing may be particularly useful in scenarios in which the receiver must process heterogeneous signals without knowing in advance which temporal characteristics are most informative.

Several issues remain to be addressed before deployment in practical open wireless environments. The current evaluation is based on simulated benchmark datasets, and some analog modulation classes remain intrinsically ambiguous under the adopted signal generation process. In addition, the parallel expert branches introduce additional inference latency compared with single branch networks. Future work will therefore evaluate MoEformer on over-the-air measured datasets and investigate hardware aware parallel execution, sparse or conditional expert routing, and structural pruning. Extending the framework to open set and few-shot modulation recognition would further improve its adaptability to previously unseen signal classes and changing deployment conditions. Overall, MoEformer provides an extensible and promising framework for adaptive temporal modeling, and can serve as a useful reference for developing more reliable and resource aware automatic modulation recognition systems.

## Figures and Tables

**Table 1:** Summary of dataset specifications and training hyperparameters.

	RadioML2016.10a	RadioML2016.10b	RadioML2018.01A
**Modulation Types **	BPSK, QPSK, 8PSK, 16QAM, 64QAM, GFSK, CPFSK, PAM4, AM-DSB, AM-SSB, WBFM	BPSK, QPSK, 8PSK, 16QAM, 64QAM, GFSK, CPFSK, PAM4, AM-DSB, WBFM	OOK, 4ASK, 8ASK, BPSK, QPSK, 8PSK, 16PSK, 32PSK, 16APSK, 32APSK, 64APSK, 128APSK, 16QAM, 32QAM, 64QAM, 128QAM, 256QAM, OQPSK, GMSK, AM-SSB-WC, AM-SSB-SC, AM-DSB-WC, AM-DSB-SC, FM
**SNR Range**	−20:2:18 dB	−20:2:18 dB	−20:2:30 dB
**Sample Format**	2×128	2×128	2×1024
**Number of Samples**	220,000	1,200,000	2,555,904
**Dataset Partition**	6:2:2	6:2:2	6:2:2
**Batch Size**	128	128	512
**Loss Function**	Cross-Entropy	Cross-Entropy	Cross-Entropy
**Optimizer**	AdamW	AdamW	AdamW
**Learning Rate**	0.001	0.001	0.001
**LR Scheduler**	ReduceLROn Plateau	ReduceLROn Plateau	ReduceLROn Plateau
**Early Stopping Patience**	15	15	15

**Table 2:** Architecture configuration of MoEformer.

Module	Layer Configuration	Other Settings
Expert LRB (×4)	Conv1D: 2→16 (k=16) DWConv1D: 16→16 (k=16) PWConv1D: 16→32 (k=1)	BN, GELU, Dropout
Gating LRB	Conv1D: 2→64 (k=5) DWConv1D: 64→64 (k=5) PWConv1D: 64→64 (k=1)	BN, GELU, GAP
Gating MLP	FC: 64→64 FC: 64→4	Softmax-based dense weighting
Feature projection	DWConv1D: 32→32 (k=3) PWConv1D: 32→128 (k=1) PWConv1D: 128→128 (k=1)	BN, GELU, Dropout
Transformer encoder (×2)	MHSA: $d_{\mathrm{model}}=128$, 4 heads CFFN: 128→32→128 DWConv1D: k=5	Pre-LN, RoPE, SiLU, Dropout
Attention pooling	Linear score: 128→1	Tanh, temporal Softmax
Classifier	FC: 128→K	*K*: number of classes

**Table 3:** Comparative performance of various models on three datasets (A: RadioML2016.10a; B: RadioML2016.10b; C: RadioML2018.01A).

Model	Dataset	Overall	SNR		Params	FLOPs	Inference Time
			−20–0 dB	>0 dB			
ResNet [14]	A	57.08%	35.30%	83.70%	96.299 K	1.7094 M	0.3701 ms
	B	63.27%	39.18%	92.71%	96.170 K	1.7093 M	0.3648 ms
	C	60.32%	18.63%	90.89%	164.184 K	25.9389 M	0.7175 ms
AMC-Net [47]	A	61.96%	38.09%	91.13%	466.144 K	46.4440 M	0.2964 ms
	B	65.05%	41.87%	93.37%	465.631 K	46.4435 M	0.2933 ms
	C	62.10%	20.75%	92.42%	2.042 M	391.0927 M	1.1110 ms
FEA-T [48]	A	59.80%	35.68%	89.28%	169.867 K	8.0864 M	2.9358 ms
	B	64.54%	40.72%	93.65%	169.834 K	8.0863 M	2.8539 ms
	C	63.37%	21.44%	**94.77%**	179.864 K	173.5749 M	8.5859 ms
MCLDNN [24]	A	60.99%	36.95%	90.38%	406.199 K	48.7460 M	0.5463 ms
	B	64.25%	40.33%	93.48%	406.070 K	48.7460 M	0.5460 ms
	C	62.23%	20.31%	92.97%	407.876 K	399.5234 M	3.4219 ms
MCformer [35]	A	60.39%	37.27%	88.64%	72.939 K	7.0056 M	0.6291 ms
	B	64.68%	41.26%	93.31%	72.810 K	7.0054 M	0.6255 ms
	C	60.24%	21.17%	88.89%	74.616 K	55.9217 M	0.7194 ms
TLDNN [49]	A	63.00%	38.80%	92.57%	245.387 K	8.0280 M	1.1173 ms
	B	65.33%	42.16%	93.64%	245.354 K	8.0280 M	1.1165 ms
	C	63.17%	21.25%	93.91%	280.760 K	23.5850 M	1.1832 ms
**MoEformer**	A	**63.74%**	**39.87%**	**92.92%**	187.728 K	14.6029 M	1.6955 ms
	B	**66.24%**	**43.72%**	**93.77%**	187.599 K	14.4737 M	1.6901 ms
	C	**64.22%**	**22.87%**	94.55%	237.269 K	25.6846 M	1.8196 ms

**Table 4:** Ablation study on multi-scale expert configurations with different temporal scale factors.

Configuration	RadioML2016.10a				RadioML2016.10b				RadioML2018.01A			
	Avg. Acc. (%)	Params (K)	FLOPs (M)	Inference Time (ms)	Avg. Acc. (%)	Params (K)	FLOPs (M)	Inference Time (ms)	Avg. Acc. (%)	Params (K)	FLOPs (M)	Inference Time (ms)
[1.0, 2.0]	62.46	184.78	14.25	1.3521	65.52	184.65	14.25	1.3603	62.26	227.06	23.66	1.4128
[0.5, 1.0, 2.0]	61.72	186.26	14.34	1.5272	65.87	186.13	14.34	1.5234	62.52	232.16	24.16	1.6008
[0.5, 0.75, 1.0, 2.0]	62.02	187.73	14.47	1.6918	**66.24**	187.60	14.47	1.6901	62.60	237.27	24.92	1.8253
[0.5, 1.0, 1.25, 2.0]	62.25	187.73	14.56	1.6921	65.93	187.60	14.56	1.6833	62.72	237.27	25.43	1.8208
[0.5, 1.0, 1.5, 2.0]	**63.74**	187.73	14.60	1.6955	65.88	187.60	14.60	1.7030	**64.22**	237.27	25.68	1.8196
[0.5, 0.75, 1.0, 1.5, 2.0]	61.91	189.20	14.73	1.8534	65.61	189.07	14.73	1.8618	62.67	242.37	26.44	2.0383

**Table 5:** Ablation study of MoEformer on three datasets (A: RadioML2016.10a; B: RadioML2016.10b; C: RadioML2018.01A).

Model	Dataset	Overall	SNR		Params	FLOPs	Inference Time
			−10–−2 dB	0–10 dB			
Uniform Expert Weighting	A	63.27%	55.56%	92.66%	177.804 K	13.9186 M	1.5419 ms
	B	65.57%	61.04%	93.34%	177.675 K	13.7894 M	1.5349 ms
	C	62.68%	29.16%	80.85%	227.345 K	20.2408 M	1.7220 ms
Single Expert (w/o MoE)	A	63.04%	55.42%	92.33%	173.580 K	13.2240 M	1.0496 ms
	B	65.53%	60.52%	93.34%	173.451 K	13.2239 M	1.0178 ms
	C	61.62%	29.15%	79.06%	212.225 K	16.1857 M	1.0820 ms
Time-Domain Alignment	A	62.96%	55.20%	91.96%	187.728 K	14.6029 M	1.4430 ms
	B	66.08%	61.84%	93.38%	187.599 K	14.4737 M	1.4613 ms
	C	63.31%	29.76%	82.22%	237.269 K	25.6846 M	1.8654 ms
**MoEformer (Full)**	A	**63.74%**	**56.85%**	**92.72%**	187.728 K	14.6029 M	1.6955 ms
	B	**66.24%**	**62.25%**	**93.42%**	187.599 K	14.4737 M	1.6901 ms
	C	**64.22%**	**31.16%**	**84.57%**	237.269 K	25.6846 M	1.8196 ms

## Data Availability

The datasets used in this study are RadioML2016.10a, RadioML2016.10b, and RadioML2018.01A. These datasets are available from the first author upon reasonable request (jiale.wang@bistu.edu.cn).
